# Sign language encodes event structure through neuromotor dynamics: motion, muscle, and meaning

**DOI:** 10.3389/fpsyg.2025.1689676

**Published:** 2025-10-09

**Authors:** Julia Krebs, Eric Harbour, Evie A. Malaia, Ronnie B. Wilbur, Julia Martetschläger, Hermann Schwameder, Dietmar Roehm

**Affiliations:** ^1^Department of Linguistics, University of Salzburg, Salzburg, Austria; ^2^Department of Kinesiology, University of Salzburg, Salzburg, Austria; ^3^Department of Speech, Language and Hearing Sciences, University of Alabama, Tuscaloosa, AL, United States; ^4^Department of Linguistics, Purdue University, West Lafayette, IN, United States; ^5^Centre for Cognitive Neuroscience (CCNS), University of Salzburg, Salzburg, Austria

**Keywords:** Austrian Sign Language, verbs, telicity, event visibility, kinematics, muscle activation, motion capture, electromyography

## Abstract

**Introduction:**

This study provides neuromotor evidence for the embodied kinematic encoding of grammatical event structure in sign language, using time-locked motion capture and surface electromyography (EMG) recordings from fluent Deaf ÖGS signers.

**Methods:**

Drawing on the Event Visibility Hypothesis, we examine how Austrian Sign Language (ÖGS) systematically distinguishes telic and atelic verbs through both visible kinematic parameters, as well as underlying muscle activation patterns.

**Results:**

We show that telic signs (those denoting bounded, goal-directed events) have shorter duration, later deceleration, lower movement variability, and distinct spectral activation in forearm and upper-arm muscles, as compared to atelic verb signs. Telic signs showed greater EMG co-contraction but lower cross-correlation than atelic verb signs, reflecting temporally precise antagonistic muscle coordination, and suggesting that grammatical contrasts in sign language are produced based on finely tuned motor control schemas.

**Discussion:**

These results directly address current challenges in embodiment research by demonstrating replicable, interpretable neuromotor correlates of linguistic structure in a visual-manual modality. By capturing how grammatical distinctions are produced by manual articulators, we contribute high-resolution empirical data and analysis methods toward understanding embodied language and linguistic motor control. In addition, our results support the linguistic interpretation that telic verb signs are morphologically marked in a way that atelic verb signs are not.

## 1 Introduction

Embodied theories of language suggest that grammatical distinctions are grounded in sensorimotor experience; however, empirical evidence at the neuromuscular level remains sparse and modality-specific. Sign languages provide a unique empirical window into embodied cognition precisely because their grammar is realized in physical movement. In this study, we quantify a specific type of embodiment - event visibility in sign languages—by demonstrating how grammatical event structure in Austrian Sign Language (ÖGS) is encoded via systematic differences in motor control of verb sign production, quantified using kinematic and electromyographic (EMG) data. Building on the Event Visibility Hypothesis (Wilbur, 2003, 2008; Wilbur et al., 2012), which links event semantics to articulatory dynamics in sign languages, we show that neuromotor coordination in signers produces a kinematic pattern that is critical for grammatical marking of verb event structure, or differentiation between telic and atelic verbs. Semantically, telic verbs describe actions with clear endpoints (similar to English verbs “arrive” or “break”), while atelic verbs describe processes that do not have natural endpoints (similar to English verbs “run” or “think”). Our data show that telic and atelic verbs differ both in spatiotemporal patterns of overt kinematics and in muscle-specific activation patterns and co-contraction strategies, suggesting deep integration between linguistic computation and articulator control. By combining motion capture and EMG in fluent Deaf signers, this study also addresses methodological gaps in the embodiment literature by offering a replicable, high-resolution paradigm for examining how linguistic and motor control systems interact in proficient signers.

For a number of unrelated sign languages it has been reported that event structure is reflected in the visual and dynamic form of verb signs, an observation which was formulated as the Event Visibility Hypothesis (Wilbur, 2003, 2008). Wilbur (2003, 2008) described that in American Sign Language (ASL) the set of verb signs denoting telic events, which have a natural endpoint (e.g. arrive), differ from the set of verb signs denoting atelic events, which lack a natural endpoint (e.g. run) in both phonological form and syllable structure. Telics are produced with a sharper ending to a stop resulting from a change of handshape aperture, a change of handshape orientation, an abrupt stop at a location in space or a contact with a body part. Atelics which lack endpoint marking are produced by a straight or curved path movement or do not show any movement at all (Wilbur, 2003, 2008).

Although such event structure is visible in different sign languages, cross-linguistic differences can be observed. For instance, whereas in ASL telics and atelics differ in the phonological structure of their lexical items, in Croatian Sign Language (HZJ) an overt morphological process is used to produce an alternation between two forms of a verb from one stem, where the verb root is signed with shorter, sharper movement for the telic form compared to its atelic form (Milković, 2011). These movement-based distinctions have been confirmed by studies using motion capture: for ASL a faster deceleration at sign end was observed for telics compared to atelics (Malaia and Wilbur, 2008, 2012), whereas for HZJ faster deceleration as well as higher peak velocity for telics compared to atelics was reported (Malaia et al., 2013). Interestingly, changes in speed (acceleration and deceleration) have been shown to be relevant for comprehension and the identification of event boundaries (start and end of an action) when viewing non-linguistic visual action (e.g., movement of individual objects, or activities like cooking or folding clothing) (Zacks et al., 2006).

It has been suggested that it is this non-linguistic ability that enables hearing non-signers to perceive these movement distinctions when they are used in telic and atelic signs and use them to classify telic/atelic verb signs in a two-choice lexical decision task (Strickland et al., 2015; Malaia and Wilbur, 2012; Krebs et al., 2023a). Non-signers appear to segment the sign language signal into visuomotor discrete events as they try to map the sign to a linguistic concept. This process might indicate the potential evolutionary pathway of co-optation of perceptual motion features into the linguistic structure of sign languages (Krebs et al., 2023a).

As in other sign languages, in ÖGS modulations of movement and dynamics mark linguistically relevant grammatical distinctions (Krebs and Fenkart, 2024), and event structure and the telic-atelic distinction is reflected in the phonological form of verb signs (Schalber, 2006; Krebs et al., under review^1^). Previous motion capture research on ÖGS shows that telic verbs are produced with higher acceleration and jerk, higher deceleration at the end of the signs, higher peak velocity and shorter duration than atelic verbs. In comparison to atelics, telic verbs displayed sign-final holds (whereby the hands are briefly held in space) that were 2.5 times longer than atelics (Krebs et al., 2021, 2024, 2023a). A previous study investigated kinematic and EMG data of one Deaf ÖGS signer producing 10 telic and 10 atelic verbs. The telic signs, which are produced with higher acceleration, jerk and deceleration at sign end, also displayed higher activation in upper arm muscles during the sign and hold interval as compared to atelics. In contrast, the repeated arm/hand movement used in the majority of the atelics, but absent in telics, displayed higher muscle activation in the forearm as compared to the telics (Krebs et al., 2023a).

This study aims to investigate the motor control that governs articulatory dynamics in sign language production used to express differences in meaning and grammar. Therefore, in the present study the production of telic and atelic signs of Austrian Sign Language (ÖGS) produced by six Deaf signers is examined using the motion capture and electromyography (EMG) methodology.

## 2 Material and methods

### 2.1 Participants

Six Deaf signers (4 F) were included in the analysis (*M* = 55 years, *SD* = 9; range = 40-64). All participants were either born Deaf or became Deaf early in life. Each was a fluent user of ÖGS, used ÖGS as their primary language in daily life, and identified as members of the Deaf community. All had a long-standing association with our research. Five participants self-reported as right-handed; one as left-handed.

### 2.2 Motion capture procedure

**Figure 1:**
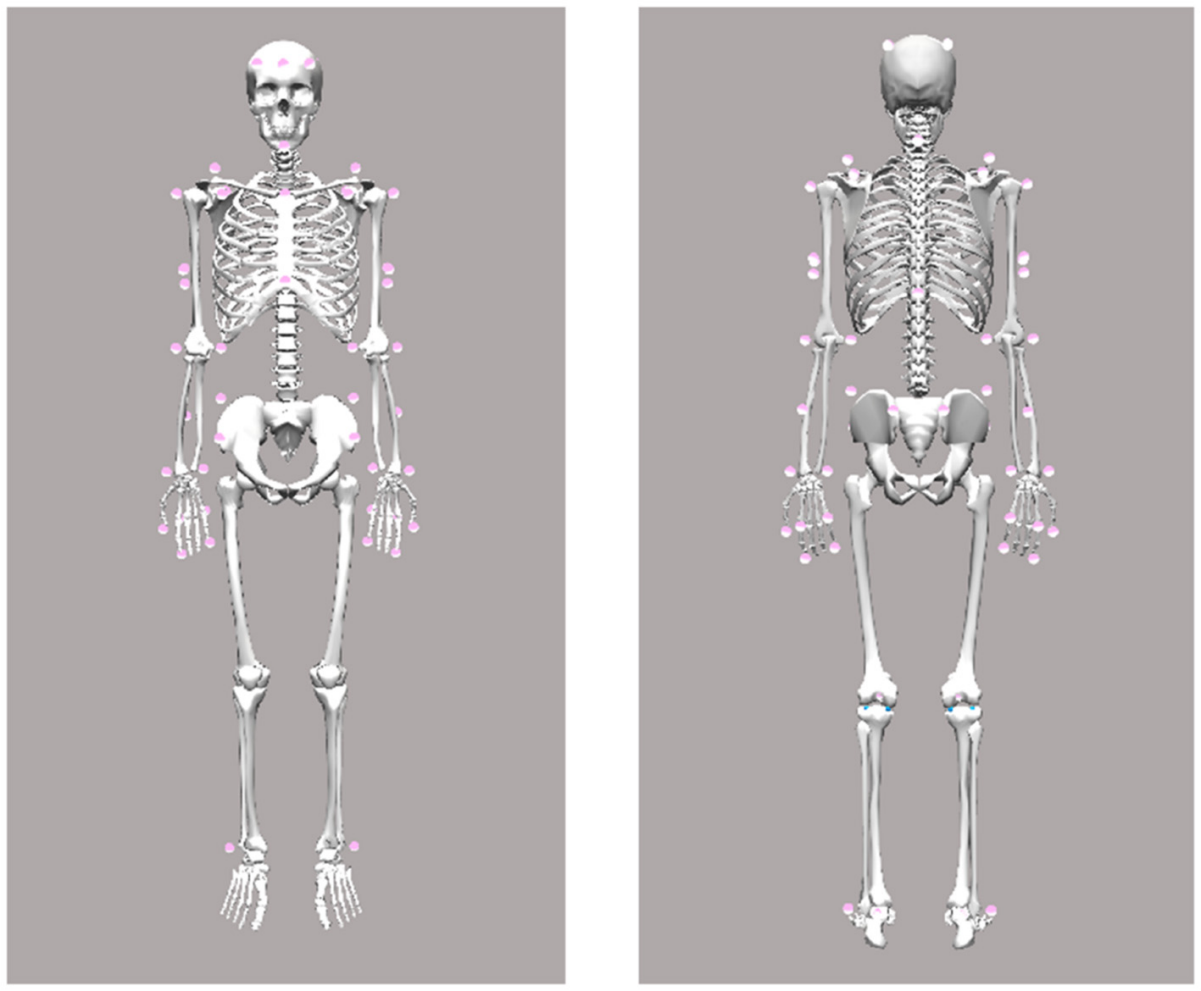
Motion capture marker set.

Body kinematics—including torso, head, and arms/hands –were recorded using a custom-designed marker set (see Figure 1) and a 12-camera infrared motion capture system (Qualisys AB, Göteborg, Sweden) operating at a sampling rate of 300 Hz. Simultaneously, a 2D video of the participant was recorded at 150 Hz and time-synchronized with the motion capture data. Marker trajectories were low-pass filtered using a second-order, zero-lag Butterworth filter with a cutoff frequency of 25 Hz. Segment positions and orientations were calculated using an inverse kinematics algorithm (V3D; C-Motion, Rockville, MD, USA). Joint centers at the wrist, elbow, and shoulder were estimated as virtual landmarks positioned midway between the lateral and medial anatomical markers (see Figure 1).

All signers started their hand/arm movement from the same resting position with the arms at the sides of the body. The start and end of the sign phase was visually set by a skilled signer using 2D video recording time-aligned to motion capture data. Sign onset was defined as the video frame when the target handshape reached the target location from where the sign movement started (Wilbur and Malaia, 2008). The sign offset was defined as the video frame when the handshape or the hand orientation of the sign changed or when the hand moved away from the final position. The dominant hand was operationalized as the one used for producing one-handed signs; in two-handed asymmetric signs, the dominant hand performs the primary articulation while the non-dominant hand serves as a passive articulator. For statistical analysis, each sign was analyzed individually, using dominant hand data per signer and per sign.

**Figure 2:**
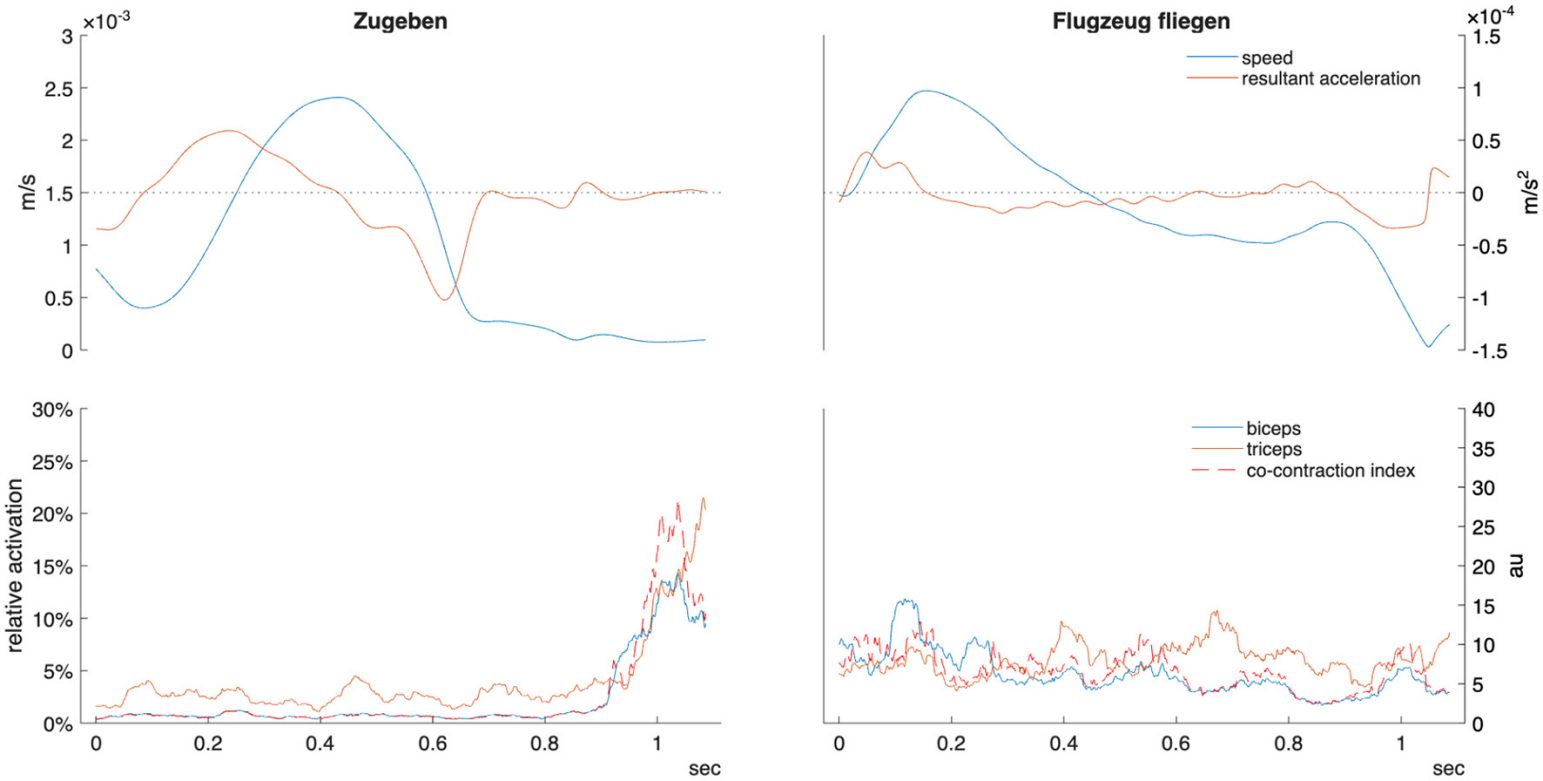
Example kinematic and EMG data for telic verb “zugeben” (confess/admit) and atelic verb “Flugzeug fliegen” (fly by plane) in one participant. Sign time displayed from manual start and end labels as detailed in the methods section. Relative activation indicates percentage of maximal voluntary contraction. Co-contraction index units are arbitrary (au). m/s, meters per second.

After trimming to sign phase, sign duration was extracted and then the position data were time-normalized to 100 points using spline interpolation. First, second, and third derivatives were calculated using MATLAB function “gradient" to obtain velocity, acceleration, and jerk, respectively (see Figure 2). Velocity data were transformed using the Euclidean norm to obtain absolute speed, from which the median and peak speed (*m*/*s*) were labelled. Resultant acceleration was also calculated, from which peak deceleration (*m*/*s*^2^) and the time to peak deceleration (0 − 100% of sign) were extracted. Peak jerk and time to peak jerk were similarly extracted from the absolute jerk vector. Movement entropy was calculated from the speed vector using the function “SampEn” with an embedded dimension m=2 and tolerance r = 0.2^*^standard deviation (Lee, 2012). Finally, movement variability was quantified using spatiotemporal index (STI). STI, which measures how consistently participants performed the same movement pattern across repetitions, was calculated for velocity and acceleration by calculating the standard deviation (SD) at every other time point (50 SDs) and then summing all SDs into a scalar value (Howell et al., 2009).

## 3 Electromyography (EMG) recording procedure

In biomechanics and kinesiology, surface electromyography (EMG) is widely employed as a non-invasive method for monitoring electrical activity at the surface of skeletal muscles. It is particularly useful for determining the onset and offset of muscle activation, as well as assessing contraction magnitude and patterns of intermuscular coordination (Schwameder and Dengg, 2021).

The EMG analysis was performed using EMG sensors (Ultium(TM) EMG, Noraxon, Scottsdale, AZ, USA) connected to surface electrodes (Ambu blue, 30 × 22 mm, Ag/AgCl). Data were collected simultaneously with the kinematic analysis through the Qualisys Track Manager (Qualisys AB, Goteborg, Sweden). EMG data were collected at 2000 Hz. EMG signals were recorded from four arm muscles: m. extensor digitorum, m. flexor digitorum, m. biceps brachii and m. triceps brachii of the dominant arm. EMG electrodes were placed on the participant's skin, which was prepared beforehand (by shaving and disinfecting the skin to remove skin scales, hair, and skin oil to get the best possible EMG signal) at specific anatomical places (e.g. thickest part of the muscle of interest) according to the recommendations of SENIAM (http://www.seniam.org/).

Participants performed maximum voluntary contraction (MVC) procedures according to best practices (Burden, 2010) by contracting against a fixed object in standardized positions (wrist 0, elbow 90 degrees flexion). They were given strong verbal encouragement to push maximally for three seconds. MVC estimation was performed as it allows for more accurate comparison of activation levels between adjacent muscles.

The power spectral density (PSD) estimate was obtained from the sign phase raw EMG signals using the function “periodogram”. Then, the mean and median frequency were extracted and the power in five distinct frequency bands was dervied for comparison (6–15, 16–25, 26–60, 61–75, and 76–140 Hz). This approach was selected as Roman-Liu and Konarska (2009) demonstrated its sensitivity and specificity to comparing different muscle contractions below 30% maximal contraction. The PSD of frequencies below 6 and above 140 Hz were also calculated in order to provide a more complete picture of the full power spectra.

EMG data were post processed using MATLAB according to best practice recommendations (Muceli and Merletti, 2024). Raw EMG data were high-pass filtered at 10Hz, low-pass filtered at 300Hz then notch filtered at 50Hz to remove power line interference. Next it was rectified and smoothed using root mean square with a moving window of 100 data points (0.05 s). MVC was extracted from the highest 1s average in accordance with current best practices (Burden, 2010). Sign phase EMG was trimmed and normalized to MVC. The mean, median, and peak (in 0.25 s windows) activation was extracted for comparison.

A co-contraction index (CCI) was calculated to approximate the degree of activation between agonist and antagonist muscles in the dominant hand (e.g., upper arm biceps and triceps) (Li et al., 2021). The formula of Rudolph et al. (2000) was adapted to consider that certain sign expressions had alternating agonist muscles within the sign. Thus, at each sample point the following formula was used:


(1) $$\begin{array}{l} \left(emg_{l}/emg_{h}\right)*\left(emg_{l}+emg_{h}\right) \end{array}$$

Where *emg*_*l*_ is the EMG signal with a lower amplitude and *emg*_*h*_ higher. Mean and peak (0.25 s window) CCI during the sign phase were retained for comparison.

### 3.1 Cross-correlation analysis

Cross-correlation analysis provides a quantitative measure of how well muscle activation patterns align with hand movement timing. The cross-correlation of each upper arm muscle (biceps and triceps) with wrist speed was estimated using the MATLAB function “xcorr” with a maximum lag of 20% sign duration and normalized output. Peak cross-correlation coefficients were extracted as the maximum absolute correlation value within the lag window. Cross-correlation values were Fisher z-transformed prior to statistical analysis to ensure normality assumptions were met, then back-transformed for reporting. Higher cross-correlation values indicate stronger temporal coupling between muscle activation and wrist kinematics.

## 4 Statistical analysis

Unless otherwise noted, all data are reported as median ± interquartile range. Mixed-effect linear models (MATLAB package fitlme) were used to examine the effect of verb type (telic or atelic) upon all kinematic and EMG variables. The model included verb type as the fixed effect, with a random intercept for nested verb type/participant. Thus, the formula was:

variable ~ verb type + (verb type | participant)

Partial eta squared effect sizes ($\eta_{p}^{2}$) were reported and interpreted as small = 0.01; medium = 0.06; and large = 0.14. P-values were adjusted using the Benjamini-Hochberg method (MATLAB package mafdr) to account for multiple comparisons.

## 5 Results

**Figure 3:**
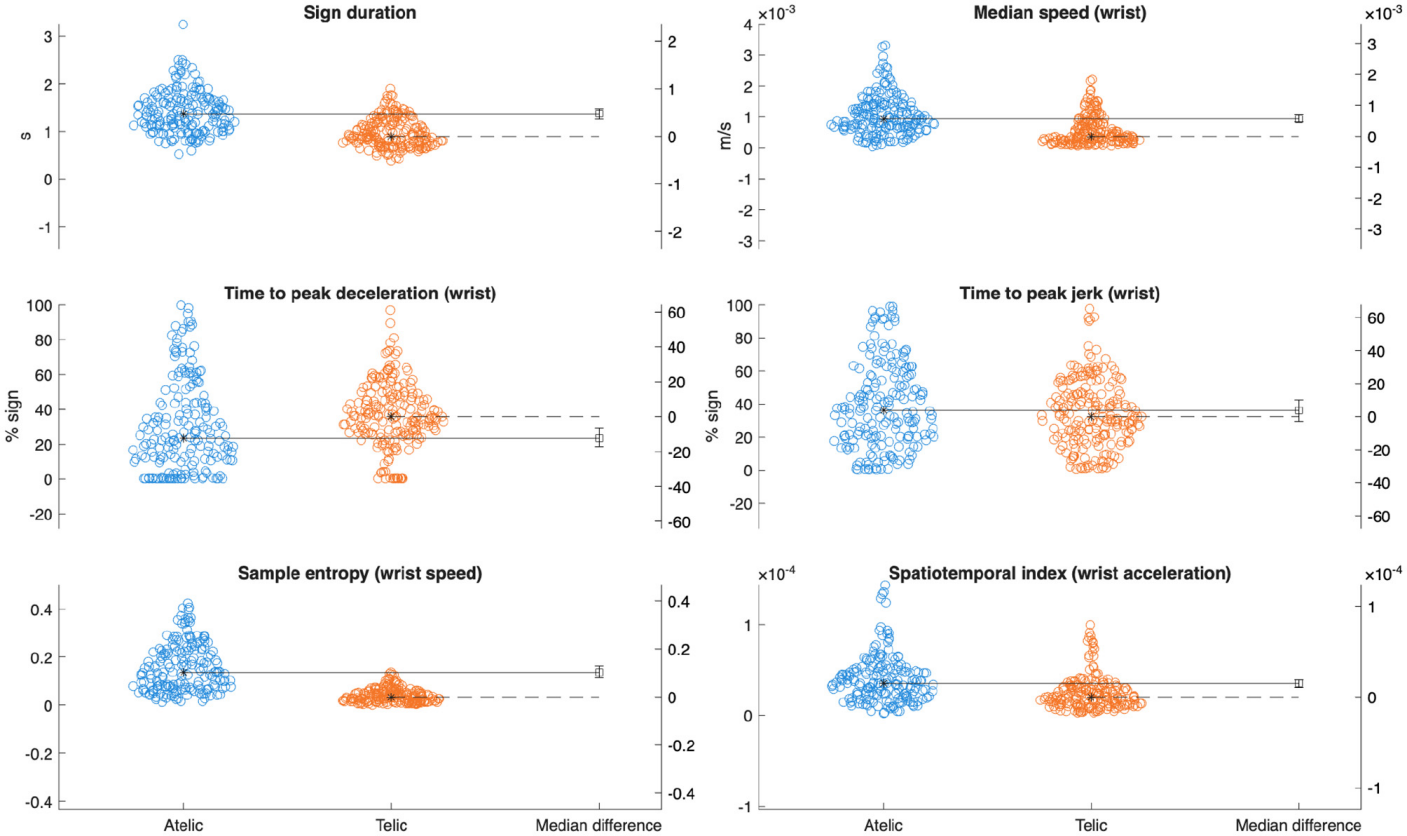
Gardner-Altman plots with median differences comparing key kinematic variables between verb classes.

A total of 390 signs (34 telic and 31 atelic verbs per participant) were collected and included in this analysis. Pooled kinematic data are displayed in Table 1. Linear mixed-effects models revealed statistically discernible differences in movement kinematics between telic and atelic verbs. Telic verbs were characterized by significantly slower average wrist speed (estimate [Est] = −0.00047 m/s, SE = 0.00013, *p* < 0.001, $\eta_{p}^{2}$ = 0.034), shorter sign duration (Est = −0.44 s, SE = 0.083, p < 0.001, $\eta_{p}^{2}=$ 0.070), and later timing of peak deceleration (Est = 6.32%, SE = 2.32, *p* = 0.012, $\eta_{p}^{2}=$ 0.019) compared to atelic verbs (see Figure 3). Telic verbs also showed lower sample entropy (Est = −0.12, SE = 0.013, *p* < 0.001, $\eta_{p}^{2}=$ 0.17) and lower spatiotemporal indices for velocity and acceleration (both *p* < 0.001, $\eta_{p}^{2}=$ 0.035 and 0.070, respectively), reflecting more regular and less variable movement patterns. These results are consistent with telic verbs involving brief, controlled, and well-structured movement profiles.

**Table 1:** Comparison of kinematic measures between telic and atelic signs.

**Measure**	**Telics median ±IQR**	**Atelics median ±IQR**	**LME estimate**	**SE**	***p* (adjusted)**	** $\eta_{p}^{2}$ **
Median speed (m/s)	3.8 × 10^−4^±5.7 × 10^−4^	9.4 × 10^−4^±7.7 × 10^−4^	−4.7 × 10^−4^	1.3 × 10^−4^	<0.001	0.034
Peak speed (m/s)	3.3 × 10^−3^±1.9 × 10^−3^	2.1 × 10^−3^±1.2 × 10^−3^	4.6 × 10^−3^	3.5 × 10^−3^	0.100	0.005
Duration (s)	0.90 ± 0.43	1.37 ± 0.60	−0.44	0.083	<0.001	0.070
Sample entropy	0.034 ± 0.040	0.14 ± 0.16	−0.12	0.013	<0.001	0.169
Peak deceleration (m/s^2^)	−9.5 × 10^−5^±8.9 × 10^−5^	−6.5 × 10^−5^±4.9 × 10^−5^	−7.1 × 10^−5^	5.1 × 10^−5^	0.097	0.005
Time to peak deceleration (% sign)	35.85 ± 21.52	23.32 ± 33.92	6.32	2.32	0.012	0.019
Peak jerk (m/s^3^)	1.4 × 10^−5^±1.6 × 10^−5^	1.3 × 10^−5^±3.3 × 10^−5^	6.5 × 10^−5^	8.8 × 10^−5^	0.190	0.001
Time to peak jerk (% sign)	32.41 ± 26.30	36.09 ± 37.8	−4.71	3.52	0.102	0.038
Spatiotemporal index (speed)	3.0 × 10^−4^±3.0 × 10^−4^	4.2 × 10^−4^±3.5 × 10^−4^	−1.3 × 10^−4^	3.4 × 10^−5^	<0.001	0.035
Spatiotemporal index (acceleration)	2.0 × 10^−5^±2.0 × 10^−5^	3.0 × 10^−5^±3.0 × 10^−5^	−1.5 × 10^−5^	3.4 × 10^−6^	<0.001	0.083

Wrist extensors showed increased mean activation during telic verbs (Est = 1.39% MVC, SE = 0.68, *p* = 0.035, $\eta_{p}^{2}$ = 0.011) (see Table 2). Wrist flexors also had elevated mean activation and peak activation in telic verbs (mean: Est = 0.92% MVC, SE = 0.39, *p* = 0.026, $\eta_{p}^{2}$ = 0.014; peak: Est = 2.24% MVC, SE = 0.79, *p* = 0.010, $\eta_{p}^{2}$ = 0.020). The triceps displayed significant increases in mean (Est = 1.14% MVC, SE = 0.40, *p* = 0.010, $\eta_{p}^{2}$ = 0.020), median (Est = 0.64% MVC, SE = 0.29, *p* = 0.031, $\eta_{p}^{2}$ = 0.012), and peak activation (Est = 2.35% MVC, SE = 0.68, *p* = 0.002, $\eta_{p}^{2}$ = 0.030) for telic verbs.

**Table 2:** Comparison of EMG measures between telic and atelic signs.

**Measure**	**Telics median ±IQR**	**Atelics median ±IQR**	**LME estimate**	**SE**	***p* (adjusted)**	** $\eta_{p}^{2}$ **
**Co-contraction**						
Forearm CCI (mean)	6.12 ± 5.2	5.14 ± 4.46	1.08	0.43	0.016	0.017
Forearm CCI (peak)	9.78 ± 9.23	8.13 ± 7.05	2.13	0.83	0.016	0.017
Upper arm CCI (mean)	4.18 ± 3.66	3.63 ± 3.43	0.61	0.29	0.034	0.014
Upper arm CCI (peak)	5.64 ± 4.62	5.46 ± 4.68	0.79	0.47	0.069	0.009
**Wrist extensors**						
Mean activation (% MVC)	10.17 ± 8.57	8.91 ± 8.04	1.39	0.68	0.035	0.011
Peak activation	14.39 ± 15.30	12.59 ± 10.46	3.07	1.07	0.010	0.021
**Wrist flexors**						
Mean activation	4.73 ± 4.67	4.10 ± 3.36	0.92	0.39	0.026	0.014
Peak activation	7.45 ± 8.90	6.18 ± 5.10	2.24	0.79	0.010	0.020
**Biceps**						
Mean activation	3.63 ± 3.18	4.03 ± 3.58	−0.24	0.45	0.224	0.001
Peak activation	5.54 ± 5.07	6.41 ± 5.19	−0.41	0.65	0.213	0.001
**Triceps**						
Mean activation	3.59 ± 3.92	2.90 ± 3.07	1.14	0.40	0.010	0.020
Peak activation	5.64 ± 6.13	4.25 ± 3.96	2.35	0.68	0.002	0.030
**Cross-correlation**						
Wrist ∝ biceps	0.81 ± 0.14	0.85 ± 0.08	−0.051	0.011	<0.001	0.056
Wrist ∝ triceps	0.82 ± 0.11	0.86 ± 0.07	−0.048	0.011	<0.001	0.050

**Figure 4:**
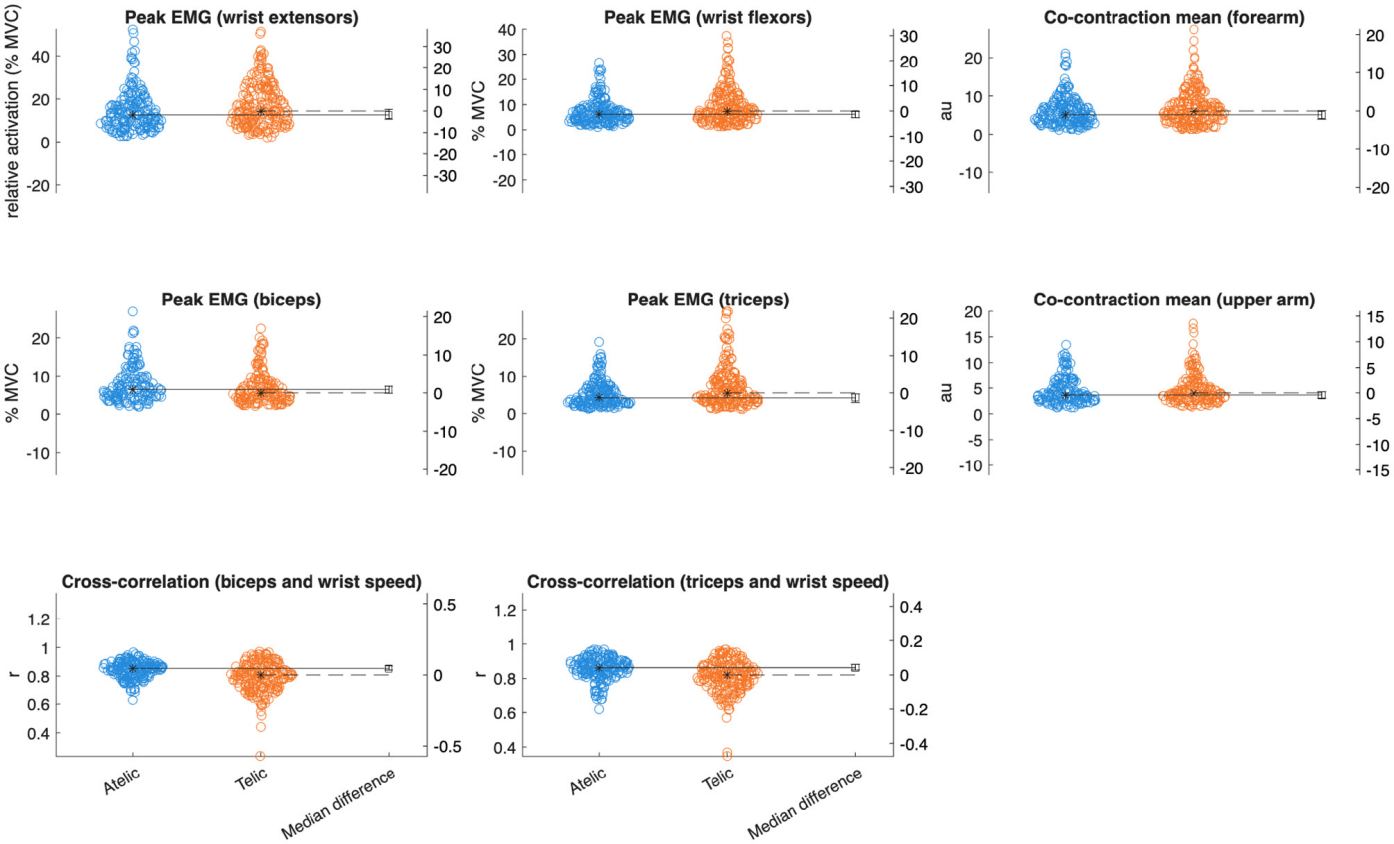
Gardner-Altman plots with median differences comparing key EMG variables between verb classes.

**Figure 5:**
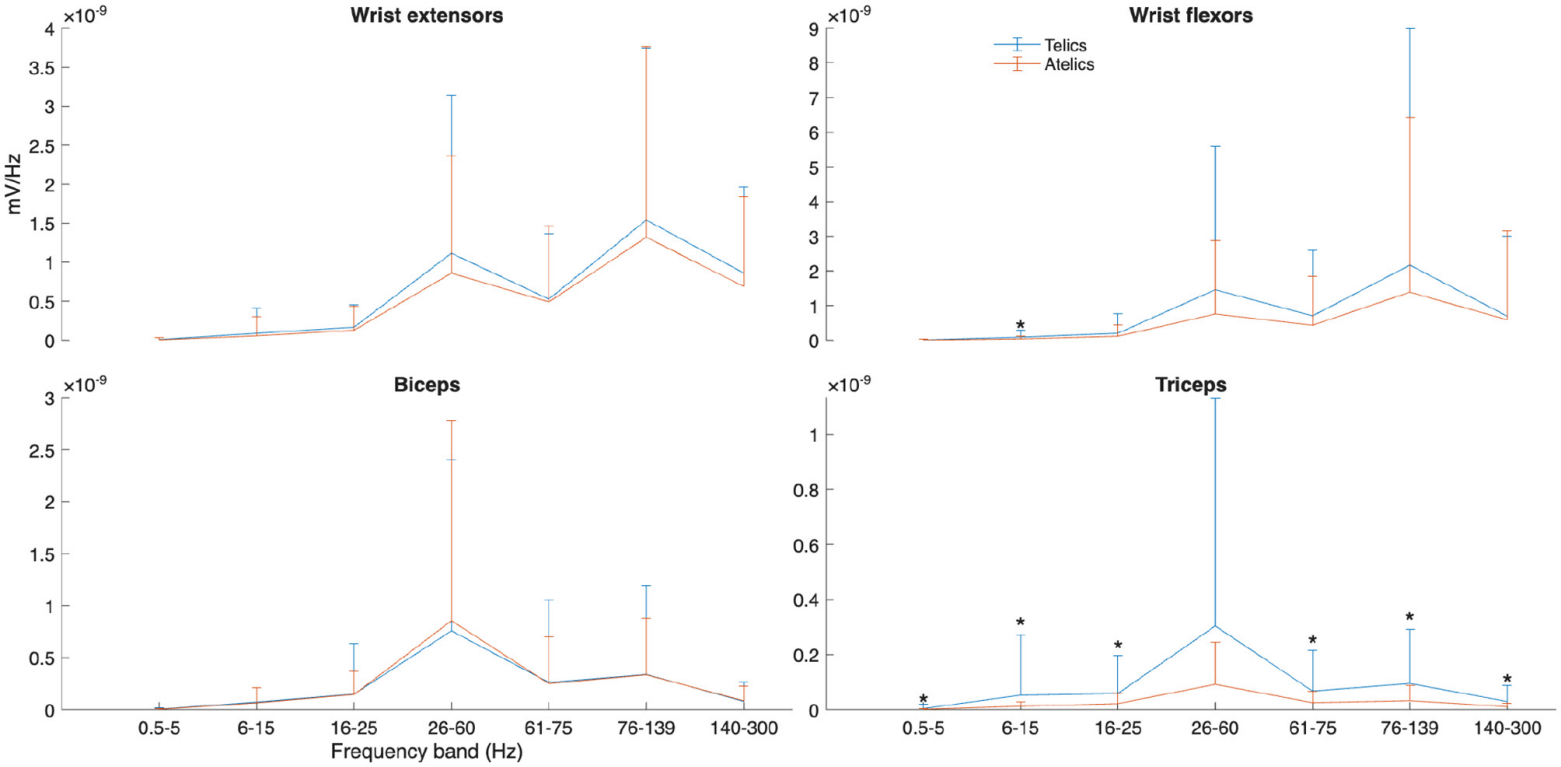
Power spectral density (PSD) of selected muscles and frequency bands for telic and atelic verbs between all participants. Significant differences between verb types at each frequency band are marked with ^*^.

PSD analyses revealed nuanced spectral differences between verb types, particularly in the triceps muscle. Telic verbs exhibited significantly higher spectral power across multiple frequency bands in the triceps, including 6–15 Hz, 16–26 Hz, 60–75 Hz, and 76–140 Hz frequency ranges (all *p* < 0.05, $\eta_{p}^{2}$ = 0.011–0.012) (see Figure 4). Wrist flexors had significantly greater power only in the 6–15 Hz band during telic verbs (*p* = 0.029, $\eta_{p}^{2}$ = 0.013), while the wrist extensors and biceps did not show significant PSD differences (see Figure 5).

Analysis of forearm and upper arm muscle co-contraction indices (CCI) indicated significantly greater mean and peak co-contraction in telic verbs compared to atelics (forearm mean CCI: Est = 1.08, SE = 0.43, *p* = 0.016, $\eta_{p}^{2}$ = 0.017; forearm peak CCI: Est = 2.13, SE = 0.83, *p* = 0.016, $\eta_{p}^{2}$ = 0.017; upper arm mean CCI: Est = 0.61, SE = 0.29, p = 0.034, $\eta_{p}^{2}$ = 0.014). This suggests greater involvement of antagonist muscle stiffness and joint stabilization during the production of telic verbs.

Cross-correlation between wrist kinematics and upper arm muscle activation differed significantly between sign types. Telic signs showed lower wrist-biceps coupling (r = 0.81 ± 0.14) compared to atelic signs (r = 0.85 ± 0.08, *p* < 0.001, $\eta_{p}^{2}$ = 0.056). Similarly, wrist-triceps coupling was lower in telic signs (r = 0.82 ± 0.11) than atelic signs (r = 0.86 ± 0.07, p < 0.001, $\eta_{p}^{2}$ = 0.050).

## 6 Discussion

This study demonstrated that the distinction between telic and atelic verbs in ÖGS is robustly encoded both kinematically and electromyographically. Telic signs are produced with lower average velocity (linked to “hold” phases within the sign), shorter duration, deceleration starting later in the movement trajectory, and much lower motion variability–consistent with a more bounded, goal-oriented motor profile (as exemplified in Figure 2). Alongside these kinematic differences, EMG analyses revealed muscle-specific activation patterns: the triceps and forearm muscles have greater activation during telic movements, in addition to which the increased low-frequency PSD (6–15 Hz) for wrist flexors and triceps within the telic verbs indicates greater common neural drive and motor unit synchronization during goal-directed movements. These findings are consistent with prior literature on EMG coherence (Castronovo et al., 2015; McManus et al., 2019).

The presence of event-marking signature patterns in sign production parallels related neural findings showing differential activation patterns for perception of telic vs. atelic verbs in sign languages (Malaia and Wilbur, 2008; Krebs et al., 2023b). That these perceptual and production findings converge suggests that the kinematic and muscle activation profiles documented here may be a part of more general cortical mechanisms for linguistically representing event boundaries.

If so, it is unsurprising that the kinematic findings for ÖGS are consistent with findings for other sign languages. Telic signs are produced with motion profiles to indicate their end-points in space and time more clearly (as compared to atelic signs), ensuring that they are distinctly visible, that is, that telicity is linguistically marked on the sign production. EMG results demonstrate how these kinematic profiles of linguistic marking of event structure in ÖGS verbs emerge from distinct muscle activation patterns—especially in frequency bands associated with low-intensity, coordinated contractions. At the neuromuscular level, increased antagonist co-contraction ($\eta_{p}^{2}$ = 0.009–0.017) and elevated muscle activations, particularly in the wrist and triceps, suggest enhanced joint stability and force control during telic actions. The power spectral density results further underscore these differences: greater PSD in low frequency bands (6–15 Hz) aligns with increased common drive to motor units, reflecting more synchronized corticospinal input during the precise control of telic movements (Castronovo et al., 2015). Specifically, the prominence of PSD increases in the triceps across a wide frequency range ($\eta_{p}^{2}$ = 0.009–0.012) suggests a critical role for this muscle in executing and terminating goal-specific arm movements. The wrist flexors' PSD increase limited to the 6–15 Hz band may indicate modulation of fine control signals rather than increased force output per se, consistent with their role in postural stabilization during grasp and manipulation phases (Laine and Valero-Cuevas, 2017).

**Figure 6:**
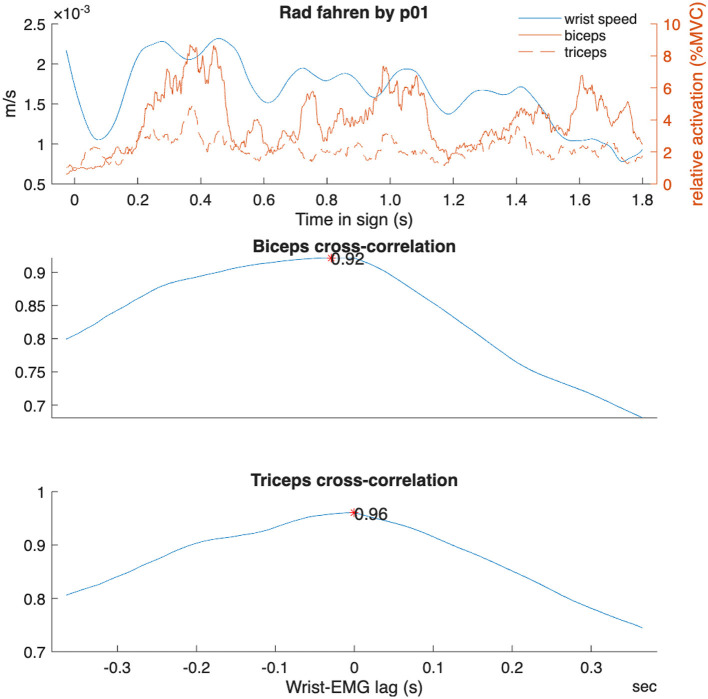
Example cross-correlation analysis for one atelic verb “Rad fahren” (to ride a bike) for one participant.

The much lower cross-correlation values in the telic verbs ($\eta_{p}^{2}$ = 0.050–0.056) may reflect the overall reduced movement combined with higher co-contraction compared to atelic signs. Atelic signs likely require more synergistic coordination across sign repetitions to maintain smooth, continuous movement (see Figure 6); furthermore, the longer average sign duration for atelic verbs might contribute to the higher cross-correlation values observed since extended signing movement demands coordinated activation, stability, and efficiency. It is also possible that wrist movement in telic verbs is driven less by elbow flexion/extension (biceps and triceps actions, respectively) and more by shoulder external or internal rotation. This would be visible in more lateral, less vertical hand movements and may be supported by the greater wrist flexor/extensor activity in telic forms ($\eta_{p}^{2}$ = 0.011-0.021); however, this was not directly tested within this study. Finally, since several of the telic verbs utilize two hands to mark the endpoint, there may be a greater role of muscles in the non-dominant arm affecting dominant wrist movement. This analysis opens new possibilities for understanding biomechanical patterns and movement efficiency during extended signing.

Overall, these results support a hierarchical motor control framework where telic verbs require increased neuromuscular precision, synchronization, and stability to achieve defined motion endpoints. This complements findings on motor unit synchronization and co-contraction patterns in skilled movement control (McManus et al., 2019; Dideriksen et al., 2018).

The neuromuscular pattern for event structure marking contrasts with the encoding of grammatical intensification in ÖGS adjectives, as documented in previous work (Krebs et al., 2025). Intensified adjectives showed consistently elevated co-contraction indices across both forearm and upper arm muscle groups, with some kinematic differences (such as prolonged time to peak deceleration in intensified adjectives). Thus, while telic verbs relied on phasic modulation, or precisely timed bursts of activation, the production of intensified adjectives appears to rely on tension, i.e. sustained co-contraction to increase joint stiffness and motion control without altering spatiotemporal trajectory. This shows that ÖGS signers employ differentiated neuromotor strategies to produce distinct grammatical feature markers. Telicity or event structure encoding was produced via control of spatiotemporal sign profiles, while adjective intensification used biomechanical stabilization (articulatory tension). This parallels functional specificity of motor control in sign language grammar of ASL and HZJ (Malaia et al., 2013), as ÖGS used both temporal precision and articulatory tension to encode linguistic contrast in the visual-manual modality.

### 6.1 Limitations

It is important to remember that the signs in the telic and atelic groups do not pair with each other. As an analogy, consider the English pair “fall” and “drop”, where “drop” can be considered the causative verb with the meaning “cause to fall”, yet “drop” and “fall” are not phonologically related to each other. The nature of this sign data, being both unpaired and collected with very sensitive measurement methods, is challenging for analysis and interpretation, requiring the removal of some outliers and the use of linear-mixed models. which are robust against such irregularities. Nevertheless, the presence of noise for both 3D motion capture and EMG likely contributes to the wide variability within these data, and might obscure meaningful differences that could be elucidated with a larger sample of participants and signs.

The EMG measurements performed here provide limited insight into actual neuromuscular control since EMG signals inherently contain a neural and peripheral component. Decomposition of these components is possible with intramuscular or high-density surface EMG, but these are not appropriate for highly dynamic movements such as those in sign language (Grison et al., 2025). Hence, the results presented here can only be used for speculative conclusions regarding common neural input and overall activation.

## 7 Conclusion

Considered together, kinematic and EMG results suggest that telic grammatical profile emerges not just from increased muscular effort, but from finely tuned temporal and directional coordination of muscle co-activation. The distinct kinematic profiles of telic verbs–marked by later deceleration and reduced variability—appear to be the downstream consequence of structured neuromuscular control, particularly antagonistic timing and targeted tension regulation in upper and lower arm muscles.

From the methods perspective, we provide quantitative neuromotor evidence that grammatical distinctions (in this case, telicity) are systematically encoded in the motor control of sign language verbs, with measurable differences in kinematics, muscle activation, and co-contraction strategies. Our modality fusion approach (combining high-resolution motion capture and EMG) provides empirical grounding for embodied cognition theory by conceptualizing linguistic embodiment as manifested in measurable physiological processes, and offering replicable methods for further work in signed and spoken languages.

In combination, these findings provide an understanding of the relationship between event boundary visibility, linguistic marking, and sign production. From a morphophonological perspective, telic verb signs exhibit consistent neuromotor marking characterized by: (1) temporally precise deceleration profiles occurring later in the movement trajectory, (2) reduced spatiotemporal variability reflecting hierarchical motor control, (3) increased co-contraction indices indicating enhanced joint stabilization, and (4) specific spectral power patterns in muscle activation. This convergent neuromotor signature constitutes a form of embodied morphological marking that renders telicity linguistically salient. Similar clear markings are not found on the atelic verb signs; rather, atelic verbs are characterized by greater kinematic variability and less coordinated muscle activation - the characteristics that are consistent with morphological unmarkedness. This asymmetry parallels canonical patterns in spoken language morphology, where marked forms exhibit consistent phonological modifications while unmarked forms show greater structural diversity. An analogy that may be helpful here is a comparison to singular and plural nouns in English. Singular nouns generally have nothing phonologically in common with each other. English plural nouns have an added morpheme (typically /s/ or /ız/). This parallels the way telic signs consistently use specific kinematic signatures to encode boundedness while atelic signs exhibit no such systematic motor control patterns (the only way in which the parallel doesn't quite work is that verb classes do not represent morphologically related derivational pairs). This pattern shows that sign language grammar relies on the principle of asymmetric marking found in spoken languages, instantiating it through coordinated neuromotor control of sign kinematics, parallel to neuromotor control of phonological modification in speech.

## Data Availability

The raw data supporting the conclusions of this article will be made available by the authors, without undue reservation.

## Footnotes

^1^Krebs, J., Wilbur, R. B., and Malaia, E. (under review). *Testing the Event Visibility Hypothesis in Austrian Sign Language (ÖGS*).
